# Improving Target Coverage and Organ-at-Risk Sparing in Intensity-Modulated Radiotherapy for Cervical Oesophageal Cancer Using a Simple Optimisation Method

**DOI:** 10.1371/journal.pone.0121679

**Published:** 2015-03-13

**Authors:** Jia-Yang Lu, Michael Lok-Man Cheung, Bao-Tian Huang, Li-Li Wu, Wen-Jia Xie, Zhi-Jian Chen, De-Rui Li, Liang-Xi Xie

**Affiliations:** 1 Department of Radiation Oncology, Cancer Hospital of Shantou University Medical College, Shantou, China; 2 Department of Clinical Oncology, Prince of Wales Hospital, Shatin, Hong Kong, China; Georgetown University, UNITED STATES

## Abstract

**Purpose:**

To assess the performance of a simple optimisation method for improving target coverage and organ-at-risk (OAR) sparing in intensity-modulated radiotherapy (IMRT) for cervical oesophageal cancer.

**Methods:**

For 20 selected patients, clinically acceptable original IMRT plans (Original plans) were created, and two optimisation methods were adopted to improve the plans: 1) a base dose function (BDF)-based method, in which the treatment plans were re-optimised based on the original plans, and 2) a dose-controlling structure (DCS)-based method, in which the original plans were re-optimised by assigning additional constraints for hot and cold spots. The Original, BDF-based and DCS-based plans were compared with regard to target dose homogeneity, conformity, OAR sparing, planning time and monitor units (MUs). Dosimetric verifications were performed and delivery times were recorded for the BDF-based and DCS-based plans.

**Results:**

The BDF-based plans provided significantly superior dose homogeneity and conformity compared with both the DCS-based and Original plans. The BDF-based method further reduced the doses delivered to the OARs by approximately 1–3%. The re-optimisation time was reduced by approximately 28%, but the MUs and delivery time were slightly increased. All verification tests were passed and no significant differences were found.

**Conclusion:**

The BDF-based method for the optimisation of IMRT for cervical oesophageal cancer can achieve significantly better dose distributions with better planning efficiency at the expense of slightly more MUs.

## Introduction

Oesophageal cancer is a frequently diagnosed cancer worldwide [1]. To achieve optimal tumour locoregional control and quality of life, multi-modal treatment strategies including operation, chemotherapy and radiotherapy are typically applied [2,3]. In fact, it is now standard to treat locally advanced cervical oesophageal cancer using concurrent chemoradiotherapy because of the difficulty of achieving a clear margin in surgical resection [4].

Intensity-modulated radiotherapy (IMRT) is an advanced radiotherapy technique that is performed using multiple small beams of non-uniform intensity that can generate very steep dose gradients, resulting in improved tumour control and fewer normal-tissue complications in general [5]. Many studies have shown that IMRT can minimise the trade-off between target coverage and organ-at-risk (OAR) sparing for oesophageal cancer [6–8]. Several clinical trials [9–12] have also reported that IMRT provides promising locoregional control with a low toxicity profile.

Cervical oesophageal cancer is typically treated with the IMRT technique because of the irregular shape of planning target volume (PTV) and the less dosimetric uncertainty caused by respiratory motion compared with that in the thoracic region. However, it is challenging to achieve optimal IMRT plans for cervical oesophageal cancer. Common reasons for this difficulty include the rapidly changing neck-to-shoulder anatomy and the presence of dose-limiting OARs; another important reason is the dose discrepancy between optimiser plans and finally calculated plans. This discrepancy is caused by an optimisation-convergence error (OCE) that originates from the following major sources, as described by Dogan et al.: tissue heterogeneity, the buildup effect, multi-leaf collimator (MLC) modulation and the optimisation algorithm [13,14]. The OCE can lead to locally high doses (hot spots) or locally low doses (cold spots) in the final dose distributions. The OCE is especially significant in the case of cervical oesophageal cancer because the PTV typically contains air cavities, such as the trachea, as well as lung tissue and the buildup region. Although selecting the optimal arrangement and number of beams is an effective approach for improving IMRT plans [15,16], the optimal beam arrangement and number alone are not able to overcome the OCE because it is a systematic error.

Accordingly, we proposed an optimisation method to compensate for the OCE with the objective of improving the planning quality for cervical oesophageal cancer. To assess the application of this new method, the original plans were used in a longitudinal comparison to demonstrate its efficacy, and another common optimisation method was used for a lateral comparison.

## Materials and Methods

### Ethics Statement

The protocol was approved by the Ethical Commission of the Cancer Hospital of Shantou University Medical College. Because this was not a treatment-based study, our institutional review board waived the need for written informed consent from the participants. The patient information was anonymised and de-identified to protect patient confidentiality.

### Patients

We retrospectively identified twenty previously untreated patients (median age 58 years, range 41–74 years), including 3 females and 17 males, with cervical oesophageal squamous cell cancers in Stage T3-T4 and N0-N1. Tumour staging was based on the American Joint Committee on Cancer 2010 7th edition staging criteria. The patients were immobilised in head-neck-shoulder thermoplastic masks in the supine position.

### Target delineation and OAR definition

The gross tumour volume (GTV), lymph nodes (LNs), clinical target volumes (CTVs), PTVs and OARs were contoured on an Eclipse version 10.0 treatment planning system (Varian Medical Systems, Palo Alto, USA).

The GTV was determined using planning CT, MR, positron emission tomography (PET) and clinical information. Two CTVs (CTV64 and CTV54) were defined for simultaneous integrated boost IMRT. The high-risk CTV (CTV64) was contoured with superior–inferior margins of 3–4 cm and 1-cm transverse margins around the GTV and with 1-cm margins around the positive LNs. The high-risk PTV (PTV64), which was generated by adding 0.5-cm margins to the CTV64, was prescribed a 64-Gy dose (2 Gy/fraction) administered in 32 fractions. The low-risk CTV (CTV54) covered the CTV64 plus nodal basins at risk of harbouring metastatic disease, namely, the lymphatic drainage area in the bilateral supraclavicular zone and the mediastinum. The low-risk PTV (PTV54), which was generated by adding 0.5-cm margins to the CTV54, was prescribed a 54-Gy dose (1.69 Gy/fraction) administered in 32 fractions. The mean volumes of the PTV64 and PTV54 were 130.5 ± 72.5 and 321.2 ± 88.9 cubic centimetres (cc), respectively.

The OARs, including the spinal cord and lungs, were delineated on each image. The planning OAR volume (PRV) that was generated from the spinal cord plus 5-mm margins was denoted as PRV spinal cord [5].

### IMRT planning techniques and planning objectives

Five coplanar sliding-window IMRT fields of 6-MV photons from a TrueBeam (Varian Medical Systems, Palo Alto, USA) linear accelerator were generated for each plan in Eclipse. The gantry angles were evenly distributed at 216°, 288°, 0°, 72° and 144°. Dose-limiting ring structures [17] were created to form dose gradients around the PTVs. The Dose Volume Optimiser (DVO, version 10.0.28) and the Anisotropic Analytical Algorithm (AAA, version 10.0.28) were employed for optimisation and for final dose calculations, respectively. The plans were normalised to the 64-Gy prescribed dose which covered 95% of the PTV64.

The optimisation objectives for the inverse planning were to achieve 95% coverage of the PTVs at the prescribed doses with the PTV64 maximum dose ≤ 70.4 Gy while limiting the doses to the OARs within specified tolerances. The PTV coverage objectives were assigned the highest priorities, followed by the OAR sparing. The notation D_x_ represents the dose that was reached or exceeded in x of the volume. The notation V_xGy_ represents the % volume that received a dose of at least x Gy. The dose-volume constraints of the OARs were set as follows: the D_0.1cc_ of the PRV spinal cord was constrained to be < 45 Gy [6]; the lung volumes were constrained to be V_5Gy_ < 45%, V_10Gy_ < 35%, V_20Gy_ < 20% and V_30Gy_ < 10%; and the mean lung dose (MLD) was constrained to be < 15 Gy [7].

To create the original plan (Original plan), the planning objectives from a template were applied and fine-tuned until the plan was clinically acceptable. With the original planning objectives unmodified, two independent methods were utilised to improve the original plans, thereby generating two additional types of plans: 1) re-optimisation utilising the base dose function (BDF-based plan) and 2) re-optimisation using dose-controlling structures to address hot and cold spots (DCS-based plan) [17,18].

**Fig 1:**
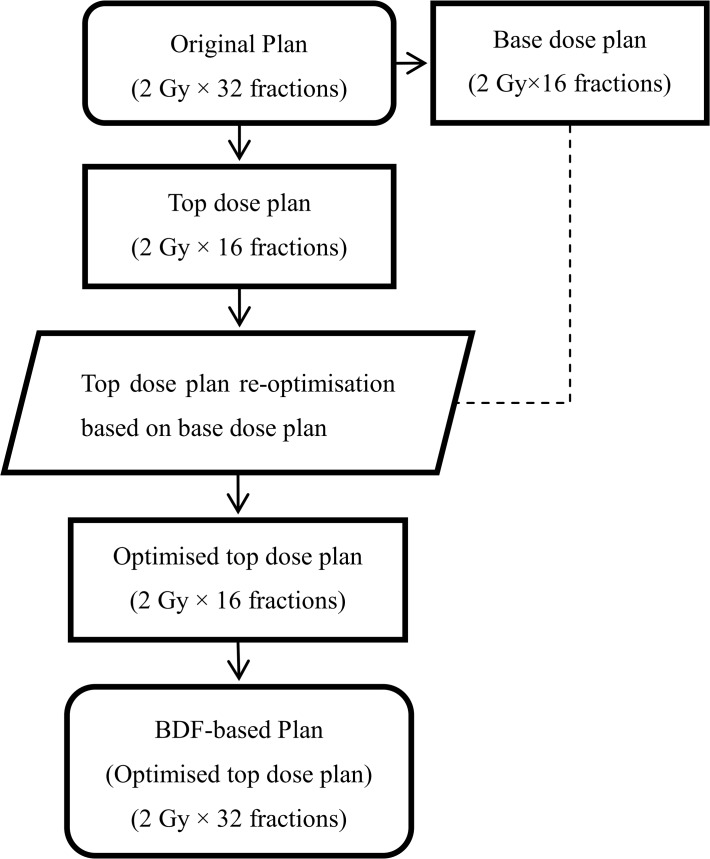
Workflow for generating a BDF-based plan for cervical oesophageal cancer.

To generate a BDF-based plan, the number of fractions of the original plan was modified to 50% of the prescribed number of fractions (from 32 to 16, in our cases) to generate a “base dose plan” with half of the total prescribed dose. Then, the base dose plan was copied to be a “top dose plan”. Afterwards, the top dose plan was re-optimised once based on the base dose plan using Eclipse’s base dose function. At this point, the prescribed dose of the plan sum (the top dose plan plus the base dose plan) was equal to the originally prescribed dose. When the final dose calculation was complete, the number of fractions of the optimised top dose plan was changed from 50% (16 fractions) to 100% (32 fractions) of the prescribed number of fractions, that is, the prescribed dose of the top dose plan was changed from a half dose to the original dose. The resulting optimised top dose plan was referred to as the BDF-based plan. This workflow is depicted in Fig. 1. To generate a DCS-based plan, the isodose of 67.2 Gy (105% of the PTV64 prescription dose) and the 45-Gy isodose in the PRV spinal cord in the original plan were converted into dose-controlling structures, and a cold-spot dose-controlling structure was generated from the PTV64 minus the prescription isodose volume (PIV). Then, the dose-controlling structures for hot and cold spots were assigned new dose objectives. Typically, for the PTV64, the upper dose objective was set to 2% lower than the prescribed dose for the PTV64 hot spots, and the lower dose objective was set to 2% higher than the prescribed dose for the cold spots. The upper dose objective was set to 40–45 Gy for the hot spots of the PRV spinal cord. After one-time re-optimisation and final dose calculation, the DCS-based plan was complete. A distributed calculation framework (DCF) was applied to accelerate the final dose calculation. The one-time re-optimisation time was defined as the time from the beginning of re-optimisation to the completion of the final dose calculation.

### Plan evaluation

According to the International Commission on Radiation Units and Measurements (ICRU) report 83 [5], D_98%_ and D_2%_ represent the near-minimal and near-maximal doses for the PTV, respectively. The homogeneity index (HI), as a measure of the target dose homogeneity, was defined as follows:

$$HI=\frac{D_{2\%}-D_{98\%}}{D_{50\%}}$$

A conformity index (CI) [19] which takes into account the overlap between target volume (TV) and PIV, was used to quantify the target dose conformity and was defined as follows:

$$CI=\frac{{\left(TV\;within\;PIV\right)}^{2}}{TV\times PIV}$$

An HI value of 0 indicated ideal homogeneity, and a CI value of 1 indicated ideal conformity. With regard to the PTV64, the D_98%_, D_2%_ and D_50%_ values were used to evaluate the cold-spot, hot-spot and median doses, respectively. For the PTV54, only the CI was used because the PTV54 was not normalised and included the PTV64. The MLD, V_5Gy_, V_10Gy_, V_20Gy_ and V_30Gy_ values were used for the lungs, and D_0.1cc_ was used to evaluate the near-maximum dose of the PRV spinal cord.

### Dosimetric verifications

The independent checking software IMSure version 3.4.1 (Standard Imaging, Middleton, USA) and a Delta^4^ diode array phantom (Scandidos, Uppsala, Sweden) were used to verify the dose accuracy of the BDF-based and DCS-based plans. The fluence for each field and the point dose for the total plan that were re-calculated using IMSure and the 3D delivered dose that was measured by the Delta^4^ phantom were compared with the corresponding values calculated in Eclipse. The fluence discrepancy and the 3D dose discrepancy were evaluated using gamma analysis with a criterion of 3%/3 mm (3% dose difference and 3 mm distance-to-agreement) [20]. The acceptable gamma pass rate was ≥ 95%, and the acceptable point-dose deviation calculated using IMSure was within ±3%. Moreover, the delivery time was recorded during the delivery of radiation to the Delta^4^ phantom.

### Statistical analysis

The differences among the BDF-based, DCS-based and Original plans were evaluated using two-sided paired t-tests in which a *P*-value of < 0.05 was considered to be statistically significant. SPSS version 19 software (SPSS, Inc., Chicago, IL, USA) was used to analyse the data.

## Results

### Target coverage, homogeneity and conformity

**Fig 2:**
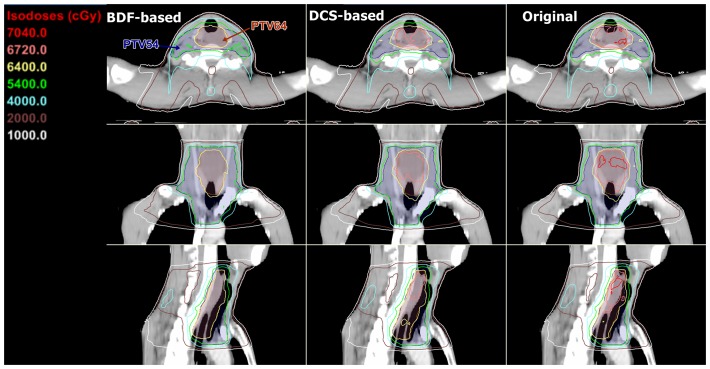
Dose distributions of the BDF-based, DCS-based and Original plans for one case.

**Fig 3:**
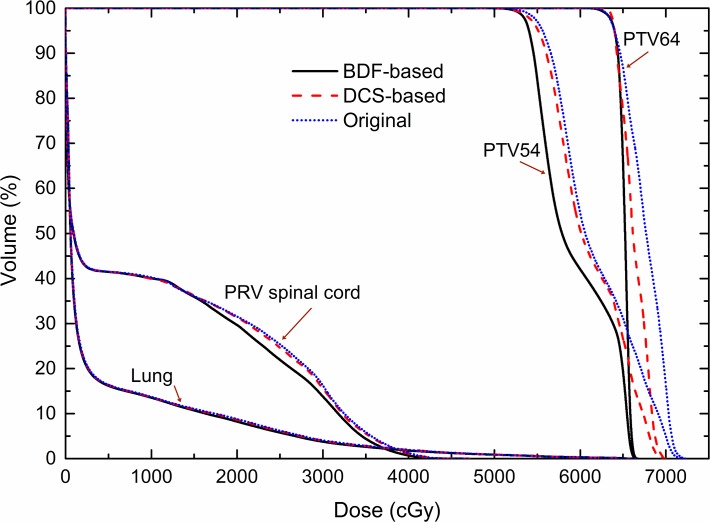
Dose-volume histograms (DVHs) of the BDF-based, DCS-based and Original plans for one case.

Table 1 summarises the target dose-volume parameters for the 3 plans. The BDF-based plans provided the best target dose distributions with respect to most parameters, whereas the DCS-based plans were inferior to the BDF-based plans but superior to the Original plans. Compared with the Original plans, the BDF-based plans demonstrated significantly improved D_2%_, D_98%_, HI and CI values for the PTV64 and an improved CI for the PTV54 by approximately 4.4%, 0.3%, 50.3%, 11.4% and 3.7%, respectively. Compared with the DCS-based plans, the BDF-based plans demonstrated better D_2%_, HI and CI values for the PTV64 and a better CI for the PTV54 by approximately 1.9%, 25.7%, 8.3%, 3.3%, respectively, as well as a comparable D_98%_ value for the PTV64. The DCS-based plans showed improvements over the Original plans in all respects except for the comparable CI for the PTV54. In the isodose distributions, significantly fewer hot spots of ≥ 105% (67.2 Gy) of the prescribed dose for the PTV64 were observed for the BDF-based plans, and the isodose lines appeared more conformal to the PTVs (Fig. 2). Besides, the dose-volume histogram (DVH) curves of the PTVs seemed far steeper for the BDF-based plans (Fig. 3).

**Table 1:** Target coverage parameters and monitor units for the BDF-based, DCS-based and Original plans.

		BDF-based	DCS-based	Original	*P*-value		
		mean ± SD	mean ± SD	mean ± SD	BDF-based vs.	BDF-based vs.	DCS-based vs.
					DCS-based	Original	Original
**PTV64**	**D**_**2%**_ **(cGy)**	6639 ± 50	6765 ± 91	6945 ± 102	0.000*	0.000*	0.000*
	**D**_**98%**_ **(cGy)**	6337 ± 21	6344 ± 22	6318 ± 16	0.246	0.003*	0.000*
	**D**_**50%**_ **(cGy)**	6537 ± 27	6591 ± 37	6688 ± 57	0.000*	0.000*	0.000*
	**HI**	0.046 ± 0.010	0.064 ± 0.014	0.094 ± 0.015	0.000*	0.000*	0.000*
	**CI**	0.874 ± 0.027	0.811 ± 0.055	0.786 ± 0.038	0.000*	0.000*	0.027*
**PTV54**	**CI**	0.828 ± 0.065	0.803 ± 0.070	0.799 ± 0.069	0.000*	0.000*	0.307
**Monitor Units**		1030.6 ± 170.8	1013.5 ± 161.5	1018.5 ± 165.6	0.004*	0.036*	0.165

BDF, base dose function; DCS, dose-controlling structure; SD, standard deviation; D_x_, dose that is reached or exceeded in x of the volume; HI, homogeneity index; CI, conformity index; PTV64, planning target volume receiving a prescribed dose of 64 Gy; PTV54, planning target volume receiving a prescribed dose of 54 Gy.

* Statistical significance.

### OAR sparing

Table 2 summarises the dose-volume parameters of the OARs for the 3 plans. In terms of the dose delivered to the PRV spinal cord, the BDF-based plans slightly reduced the D_0.1cc_ value of the PRV spinal cord by 1.1 ± 1.3% compared with the Original plans and by 2.3 ± 1.8% compared with the DCS-based plans. Concerning the dose delivered to the lungs, the BDF-based plans tended to deposit slightly lower doses. The BDF-based plans yielded MLDs which were lower by 2.7 ± 1.8% compared with the Original plans and by 2.2 ± 1.6% compared with the DCS-based plans. These results are also illustrated in Fig. 3.

**Table 2:** Dose-volume parameters of the organs at risk for the BDF-based, DCS-based and Original plans.

		BDF-based	DCS-based	Original	*P*-value		
		mean ± SD	mean ± SD	mean ± SD	BDF-based vs.	BDF-based vs.	DCS-based vs.
					**DCS-based**	**Original**	**Original**
**PRV spinal cord**	**D**_**0.1cc**_ **(cGy)**	4298 ± 164	4401 ± 220	4347 ± 183	0.000*	0.001*	0.003*
**Lung**	**D**_**mean**_ **(cGy)**	717 ± 226	735 ± 235	738 ± 236	0.000*	0.000*	0.001*
	**V**_**5Gy**_ **(%)**	31.13 ± 10.94	31.40 ± 11.06	31.45 ± 11.08	0.000*	0.000*	0.004*
	**V**_**10Gy**_ **(%)**	25.39 ± 8.80	25.79 ± 9.02	25.89 ± 9.07	0.000*	0.000*	0.002*
	**V**_**20Gy**_ **(%)**	14.28 ± 5.07	14.94 ± 5.33	15.09 ± 5.39	0.000*	0.000*	0.001*
	**V**_**30Gy**_ **(%)**	5.55 ± 2.19	5.94 ± 2.43	6.01 ± 2.46	0.000*	0.000*	0.011*

BDF, base dose function; DCS, dose-controlling structure; SD, standard deviation; D_x_, dose that is reached or exceeded in x of the volume; V_xGy_, % volume that received a dose of at least x Gy; PRV spinal cord, planning organ-at-risk volume of spinal cord.

* Statistical significance

### Efficiency of planning, dose delivery and dosimetric verifications

As shown in Table 3, the BDF-based method was more efficient than the DCS-based method with regard to the planning time. The one-time re-optimisation required 4.06 ± 0.9 and 5.68 ± 1.05 minutes for the BDF- and DCS-based plans, respectively. The BDF-based method reduced the re-optimisation time by 28.4 ± 25.1%. The MUs of the BDF-based plans were 1.7 ± 2.3% and 1.2 ± 2.4% higher than those of the DCS-based and Original plans, respectively (Table 1). The average delivery time of the BDF-based plans was 1.3 ± 1.0% more than that of the DCS-based plans (Table 3).

**Table 3:** Planning time, delivery time and verification results for the BDF-based and DCS-based plans.

		BDF-based	DCS-based	*P*-value
**One-time re-optimisation time (minute)**		4.06 ± 0.9	5.68 ± 1.05	0.000*
**Delivery time (minute)**		3.57 ± 0.24	3.53 ± 0.24	0.014*
**Gamma pass rate (Delta** ^**4**^ **) (%)**	**Total plan dose**	100.00 ± 0.00	100.00 ± 0.00	0.331
**Point-dose deviation (IMSure) (%)**	**Total plan dose**	0.09 ± 1.09	0.09 ± 1.14	1.000
**Gamma pass rate (IMSure) (%)**	**Fluence 1**	97.00 ± 1.26	97.62 ± 1.11	0.024*
	**Fluence 2**	98.11 ± 0.97	98.26 ± 0.80	0.323
	**Fluence 3**	97.75 ± 1.27	98.10 ± 1.33	0.114
	**Fluence 4**	97.72 ± 0.89	97.91 ± 0.87	0.285
	**Fluence 5**	96.65 ± 1.06	97.18 ± 0.95	0.004*

BDF, base dose function; DCS, dose-controlling structure.

* Statistical significance.

All verification tests were passed. There were no significant differences observed in terms of the gamma pass rates indicated by the Delta^4^ phantom and the point-dose deviations calculated using IMSure. The gamma pass rates of the BDF-based plans calculated using IMSure were very slightly lower than those of the DCS-based plans, but statistically significant differences were observed in only two fields. Nevertheless, these differences were so small as to be negligible.

## Discussion

To improve the therapeutic ratio and obtain optimal clinical outcomes, it is important to make full use of the IMRT technique. Our study demonstrated that the introduced BDF-based optimisation method is capable of further improving target coverage and sparing OARs.

The most obvious advantage of the BDF-based method is that it substantially improves dose homogeneity. Such improvement may be clinically beneficial for patients with cervical oesophageal cancer because the PTVs for the treatment of this type of cancer commonly include such tissues as submucosal tissue, mucosa, and bone, which may suffer complications after receiving significantly heterogeneous high doses [21]. Werner-Wasik et al. [22] have stated that a higher dose to the oesophagus may increase the risk of oesophageal toxicity, which may be life-threatening, leading to such potential consequences as perforations and fistulas [23,24]. Our study demonstrated that the BDF-based method is able to reduce hot spots by approximately 2–5% and provide excellent uniformity of the dose distribution, with an HI decrease of approximately 50%. Thus, it may reduce the risk of oesophageal toxicity.

The BDF-based method also demonstrated certain advantages with regard to target conformity and nearby-OAR sparing. It reduced the dose delivered to the spinal cord by approximately 1–3%, thus theoretically reducing the risk of radiation-induced myelitis, especially for patients with locally persistent or recurrent diseases requiring a second course of treatment. The BDF-based method also reduced the mean dose delivered to the lungs by approximately 2–3%, and reduced the V_5Gy_, V_10Gy_, V_20Gy_ and V_30Gy_ values of the lungs. It is well known that an overdose to the lungs may result in radiation-induced pneumonia, which may lead to death [25]. Many researchers have shown that the MLD, V_5Gy_, V_10Gy_ and V_20Gy_ values are useful predictors of pneumonitis [11,26]. Kumar et al. [27] have also concluded that acute and chronic pneumonitis are primarily correlated with the V_30Gy_ and V_20Gy_ values, respectively. As such, reducing all the dose-volume parameters mentioned above may reduce the risk of radiation-induced pneumonitis.

The BDF-based optimisation method is efficient in terms of treatment planning time, because only one parameter, the number of fractions, must be changed and an excellent dose distribution can be easily achieved via a simple one-time re-optimisation procedure. Improvement of the planning efficiency is beneficial for reducing the time that patients must wait until the start of treatment and thus for relieving patients’ anxieties. By contrast, the DCS-based method is time-consuming because it always requires multiple re-optimisations to further improve the plan, and furthermore, it takes time to delineate the dose-controlling structures and assign new dose constraints.

Traditionally, the base dose function is used for optimising a second plan (top dose plan), e.g., a boost plan, while taking into consideration the first plan (base dose plan), to achieve an optimal plan sum in the optimiser but not in the final calculation. However, the base dose function is used in a new way in the BDF-based method; here, it is employed to achieve an optimal second plan (top dose plan) but not a plan sum, in the final calculation but not in the optimiser. In principle, the base dose function is utilised to compensate for the OCE. When the OCE introduces a hot spot into the final calculated dose in the original plan (base dose plan), the second plan (top dose plan) will generate a cold spot in the same region to achieve a uniform summed dose. After the final dose calculation, by the effect of the OCE again, the cold-spot dose in the optimiser of the second plan (top dose plan) will approach the desired level [28].

A number of investigators have focused on possible methods or techniques for overcoming the OCE. The DCS-based optimisation method described by Süss et al. [18] and used by Xhaferllari et al. [17] is useful for compensating for the OCE, but it is only locally effective in the dose-controlling region, and it is a “trial and error” approach because the additional constraints require manual adjustments. By contrast, the BDF-based method is globally effective throughout the entire treatment region and is a systematic approach. According to the review by Broderick et al. [29] and other studies [30,31], the Direct Aperture Optimisation (DAO) technique incorporates series of deliverable MLC shapes instead of ideal intensity maps in the optimiser and thus is able to remove the error introduced by MLC modulation. Unfortunately, when it is applied in cervical oesophageal cancer, the error arising from tissue heterogeneity and the buildup effect still cannot be removed, and this error will result in hot and cold spots, according to our experience. Additionally, this technique is not available in non-DAO treatment planning systems, e.g., Eclipse version 10.0, whereas the BDF-based optimisation method is always available because a base dose function or similar base dose function is a basic feature provided in treatment planning systems for the optimisation of a second plan to achieve an optimal plan sum. Verbakel et al. [32] have overcome the error originating from tissue heterogeneity by dividing the PTV into low- and relatively high-density regions and subsequently setting a higher dose objective for the low-density region in the optimiser. This method is effective but minimises only one source of the OCE, and its complexity increases when dividing two or more PTVs.

In addition, because there have been few reports [28] regarding the BDF-based method to date, discreet dosimetric verifications should be performed to identify any error originating from the base dose function. Our verification results indicated that the BDF-based optimisation method offered adequate dosimetric accuracy, thus confirming the feasibility of this method in clinical practice.

However, the BDF-based method resulted in an increase in the MUs and delivery time by approximately 1–2%, which may slightly increase the incidence of secondary cancer [33]. The attempt to reduce the MUs remains an interesting topic that will be investigated in our future research.

## Conclusion

In this study, we evaluated the dosimetric characteristics of a simple optimisation method utilising the base dose function for cervical oesophageal cancer, and we found that this method can improve the target dose homogeneity and conformity and reduce the doses to the OARs while achieving adequate dosimetric accuracy, at the expense of slightly more MUs. Additionally, it offers improved planning efficiency. Therefore, the proposed optimisation method is recommended for incorporation into routine clinical practice for the IMRT of cervical oesophageal cancer.
